# Challenges and Opportunities in Delivering and Providing Culturally and Linguistically Tailored Cardiovascular Disease and Stroke Prevention Education: A Qualitative Exploratory Study

**DOI:** 10.1111/hex.70456

**Published:** 2025-10-03

**Authors:** Sabine M. Allida, Della Maneze, Scott William, Maree Hackett, Caleb Ferguson

**Affiliations:** ^1^ School of Nursing, Faculty of Science, Medicine and Health University of Wollongong Wollongong New South Wales Australia; ^2^ Centre for Chronic & Complex Care Research, Blacktown Hospital, Western Sydney Local Health District Blacktown New South Wales Australia; ^3^ The George Institute for Global Health, Faculty of Medicine and Health University of New South Wales Sydney New South Wales Australia; ^4^ NHMRC Centre for Research Excellence to Accelerate Innovation and Translation in Stroke Trials, Westmead New South Wales Australia; ^5^ Faculty of Health and Care University of Central Lancashire Lancashire UK

**Keywords:** CALD, cardiovascular disease, culture, language, prevention, qualitative, stroke

## Abstract

**Background:**

Cardiovascular disease (CVD) and stroke disproportionately affect culturally and linguistically diverse (CALD) communities, where access to prevention programmes is often limited by cultural, language, and systemic barriers. Understanding the experiences of healthcare professionals and nongovernment organisations in delivering and providing education to these communities is essential for developing effective, culturally tailored strategies that support equitable cardiovascular outcomes.

**Objectives:**

To explore (i) healthcare professionals', key health networks' and non‐government organisations' experiences of delivering CVD or stroke prevention education; (ii) the context i.e., barriers and enablers to delivering education; and (iii) and identify core components and ideal approach to deliver a CVD and stroke prevention education and behaviour change programme for CALD communities.

**Design:**

A qualitative exploratory study using semi‐structured interviews. Deductive thematic analysis was completed using NVivo 15.

**Setting and Participants:**

Fourteen participants were recruited and interviewed via Zoom; seven cardiovascular and stroke healthcare professionals and seven representatives from a range of federal‐ and state‐level cardiovascular and stroke nongovernment organisations and multicultural health networks.

**Results:**

Two key themes were identified: (1) challenges when providing and delivering culturally and linguistically tailored education and (2) components of an ideal education programme. Challenges in delivering CVD and stroke prevention education include the need to navigate diverse cultural health beliefs and worldviews, limited access to qualified interpreters and a lack of culturally relevant and translated resources. An ideal education programme was considered one that is simple, evidence‐based, culturally appropriate, and delivered in accessible, preferred formats. Key to sustained behaviour change beyond the programme are family and community engagement, the use of codesign approach to foster ownership and relevance from the inception, trust‐building, and ongoing reinforcement of educational content through reminders.

**Discussion and Conclusion:**

While systemic barriers such as interpreter shortages and limited funding persist, engaging families, empowering individuals, tailoring delivery methods, and embracing community‐specific strategies offer clear pathways for improving engagement and reach of CVD and stroke prevention education. Incorporating these components, particularly through codesign, will be essential in ensuring equitable cardiovascular outcomes for people from CALD communities.

**Patient or Public Contribution:**

Adults with lived experience of cardiovascular disease and stroke were engaged during the conceptualisation of this study through informal discussions. Clinicians and representatives from end‐user organisations were involved as participants in the study.

## 1 Introduction

Cardiovascular disease (CVD) and stroke remain the leading causes of morbidity and mortality worldwide [1], with significant disparities in health outcomes among culturally and linguistically diverse (CALD) communities [2, 3]. CALD is a general term that refers to communities with diverse languages, ethnicities, cultural practices, social systems, and religious beliefs [4]. In Australia, those who were born in a non‐English speaking country have higher prevalence of CVD and stroke compared with the nonindigenous Australian‐born population [5]. Disparities in health outcomes in these communities are often exacerbated by a variety of factors, including lower socioeconomic status and health literacy more so than in the general population [4], and cultural beliefs about health and illness that may differ from Western biomedical models [6].

Healthcare professionals provide early diagnosis, risk factor management, and promote healthy lifestyle choices to prevent CVD and stroke [7]. They play a critical role in secondary prevention, helping individuals manage existing CVD and stroke risk through education and behaviour change strategies. It is equally important to consider the organisational context in which CVD and stroke prevention education is delivered. Staff in public health agencies, nongovernment organisations (NGOs), and community health centres, play a pivotal role in delivering educational initiatives for CALD communities [8]. Understanding their experiences may help identify gaps in current approaches.

A key challenge in delivering effective CVD and stroke prevention education is identifying which core components and delivery methods are most effective across diverse cultural contexts. While existing CVD and stroke prevention education often includes risk factor awareness, lifestyle modification strategies, and access to resources for behaviour change [9], there is limited understanding of how cultural beliefs and practices influence the way preventative health messages are interpreted and responded to [10, 11] and how to meaningfully incorporate such cultural perspectives into prevention programmes. It also remains unclear which method of delivery adequately meets the communication needs, cultural preferences, and health literacy of CALD communities [12].

In this manuscript we present findings from the first phase of a three‐phase study which aims to codesign an educational behaviour change programme for CALD communities to prevent CVD and stroke. The objectives of phase one were to [1]: explore healthcare professionals' and staff working with/at key NGOs and multicultural health networks' experiences of delivering CVD or stroke prevention education [2]; explore the context i.e., barriers and enablers to delivering education, and [3] identify core components and ideal mode of delivery of an education and behaviour change programme for CALD communities.

## 2 Methods

### 2.1 Design

An exploratory qualitative research study using semi‐structured interviews. This study is reported in line with the Consolidated Criteria for Reporting Qualitative Research (COREQ) checklist [13] (see Supporting Information).

### 2.2 Participants, Setting and Recruitment

Purposive and snowball sampling were used to recruit participants in the study. Eligible participants included healthcare professionals (cardiologists, stroke neurologists, general practitioners, nurses, and allied health professionals) caring for people from CALD communities and representatives from key organisations who work with and/or develop or facilitate CVD or stroke prevention education and behaviour change programmes for CALD Australians. Participants had to be willing and able to attend a 60–90 min semi‐structured interview.

Healthcare professionals were invited to participate via email distribution lists and flyers circulated through a two‐site local health district general hospital, integrated with a community health centre in Western Sydney, New South Wales. Invitations were also distributed via newsletters of professional bodies such as the Australasian Cardiovascular Nursing College (https://www.acnc.net.au/), Cardiac Society of Australia and New Zealand (https://www.csanz.edu.au/), Acute Stroke Nurses Education Network (https://asnen.org/) and the Agency for Clinical Innovation Stroke Network (https://aci.health.nsw.gov.au/networks/stroke). Key cardiovascular and stroke NGOs and multicultural health networks were contacted to distribute the study invitation and flyers to relevant employees via email. These included health education or promotion officers and community engagement coordinators or managers who are involved in developing and/or delivering CVD and stroke prevention programmes to CALD communities. Those interested were sent a link to the study‐specific information sheet, e‐consent form and demographics survey on REDCap hosted by the New South Wales (Australia), Office of Health and Medical Research [14, 15]. The survey captured sex, profession, years of experience, qualification and ethnicity.

### 2.3 Ethical Considerations

The study was approved by the Western Sydney Local Health District (WSLHD) Human Research Ethics Committee, New South Wales, Australia (ETH01328; 31 July 2023). All participants were provided with study details with emphasis on the voluntary nature of study participation, the removal of all identifiers and anonymisation of all information. All participants provided electronic informed consent to participate.

### 2.4 Data Collection

One‐to‐one semi‐structured interviews were conducted via videoconference by two trained qualitative researcher (SA and DM) between December 2023 and March 2024. SA is a second‐generation CALD Australian with more than 6 years of experience as a cardiovascular/stroke researcher. DM is a first‐generation CALD migrant and overseas‐qualified medical doctor with 20 years of experience as a multicultural health promotion officer in the South Western Sydney Local Health District, where 35% of the population are born in a non‐English speaking country and 55% speak a language other than English at home. The interviews were audio recorded and transcribed verbatim by Pacific Transcription. Semi‐structured interview guides were informed by existing literature and developed based on the research teams' experience of working with multicultural communities. Refer to Tables 1 and 2 for the interview guides. Data collection continued until no new concepts were found, and saturation was observed [16]. Both researchers kept notes and shared observations with each other which were used to develop the final themes.

**Table 1:** Interview guide for health professionals.

Interview Guide
1. What is your experience of educating culturally and linguistically diverse (CALD) patients about their risk of stroke or cardiovascular disease? 2. What do you think are the unmet educational needs of CALD patients? 3. What do you think would help increase self‐management of hypertension, hyperlipidemia, diabetes, and obesity (risk factors for stroke or cardiovascular disease) amongst CALD patients? 4. How are CALD patients currently educated about managing their risk factors for stroke or cardiovascular disease? 5. What resources are available to support/educate CALD patients in managing their risk factors for stroke or cardiovascular disease? 6. What do you think are the barriers and facilitators to the uptake of these resources? 7. What do you think are important things for CALD patients to know about when managing their risk factors for stroke or cardiovascular disease? 8. How do you think CALD patients would like to receive cardiovascular disease and stroke prevention education? a. What mode of delivery would work best? b. At what time point? 9. What would ‘the ideal’ education programme/model look like? 10. How do you think we can improve the provision of cardiovascular disease and stroke prevention education and support for people from CALD communities?

**Table 2:** Interview guide for community and charity organisations.

Interview Guide
1. What are your key insights in the current landscape of educating people from culturally and linguistically diverse (CALD) communities about stroke or cardiovascular disease and strategies for risk factor reduction? 2. Is it done well? a. If yes, why? b. If no, why not? 3. How could it be improved? 4. What do you think are the unmet educational needs of people from CALD communities? 5. How should cardiovascular disease or stroke prevention education be delivered to these communities? a. By whom and when? 6. What do you think are the core components of cardiovascular disease and stroke prevention education? a. What are the most important topic areas to address? 7. If you had the opportunity to design ‘the ideal’ cardiovascular disease or stroke prevention education programme for CALD communities, what would this look like? a. What would this include?

### 2.5 Data Analysis

Thematic data analysis following Braun and Clarke's approach [17] was used to generate insights into participants' experiences within their broader sociocultural context. A deductive approach was used to ensure that the coding produced themes that were aligned with the study objectives. Two authors experienced in qualitative analysis (SA and SW) independently read and coded all transcripts using QSR NVivo 15 [18]. The codes were reviewed to identify patterns and related ideas grouped into themes. Themes were reviewed and refined iteratively. Any discrepancies were resolved through discussion with a third author (DM).

Member checking (sharing findings with participants) was conducted to minimise researcher bias and ensure trustworthiness of the results. All participants were emailed a summary report of the findings, including themes, sub‐themes and supporting quotes, and were invited to provide feedback on the interpretation of the results and whether their perspectives had been accurately captured and presented. After 6 weeks, only three participants responded via email, and all indicated agreement with the interpretation.

## 3 Results

### 3.1 Participant Characteristics

Interviews were conducted with seven clinicians and seven representatives from a range of federal‐ and state‐level cardiovascular and stroke NGOs and multicultural health networks. The clinician cohort comprised of cardiology and stroke clinical nurse consultants and allied health professionals with ≥ 10 years of experience. Representatives from key organisations were multicultural health promotion officers and community engagement coordinators/managers. There were 13 women and one man from the states of New South Wales, Victoria and Queensland. Six of the participants were from a CALD background. To prevent identification, the sex and ethnicity of the people whose quotes are presented, is not reported. More demographic details are presented in (Table 3). The average duration of the interviews was 47 min (range 35–71).

**Table 3:** General demographic characteristics of interview participants.

Interview #	Occupation	Years of experience	Institution
1	Community engagement coordinator	0–5 years	NGO
2	Physiotherapist	6–9 years	Public hospital
3	Speech pathologist	10–15 years	Public hospital
4	Registered nurse	> 20 years	Public hospital
5	Cardiology clinical nurse consultant	6–9 years	Public hospital
6	Stroke clinical nurse consultant	6–9 years	Public hospital
7	Registered nurse	10–15 years	Public hospital
8	Occupational therapist	10–15 years	Public hospital
9	Community engagement manager	> 20 years	NGO
10	Health promotion officer	16–20 years	Multicultural health network
11	Health promotion officer	10–15 years	Multicultural health network
12	Health promotion manager	> 20 years	Multicultural health network
13	Health promotion officer	6–9 years	Multicultural health network
14	Aged Care team supervisor	6–9 years	NGO

Abbreviation: NGO, nongovernment organisation.

### 3.2 Themes

Two themes were identified [1]: Challenges in providing and delivering culturally and linguistically tailored education and [2] Components of an ideal education programme. Each theme has four to six subthemes (Table 4).

**Table 4:** Study themes and subthemes.

No	Theme	Sub‐themes
1	Challenges when providing and delivering culturally and linguistically tailored education	Limited understanding of cultural health beliefs and worldviews
		Systemic barriers to cultural competence
		Limited availability and concerns about interpreter quality
		Lack of culturally relevant and translated resources
2	Components of an ideal educational programme	Culturally responsive education
		Accessible formats and preferred method of delivery
		Simple and evidence‐based
		Ongoing reinforcement of educational content
		Better engagement of family, caregivers and community
		Codesign is key to effective education

### 3.3 Challenges When Providing and Delivering Culturally and Linguistically Tailored Education

i. **Limited understanding of cultural health beliefs and worldviews**
  Participants highlighted the multifaceted complexity of delivering CVD and stroke prevention education in culturally and linguistically diverse contexts:
  You might have the same principles, but it's always more complex when you're engaging with an audience that have a whole different way of looking at their life, the way that they live, their relationship with their community, their beliefs, and even their relationship with the medical community and the health community.
  – Participant 9, Community engagement manager, NGO
  A common challenge was when healthcare professionals or community engagement coordinators delivering CVD and stroke prevention education have limited cultural understanding or confidence. Participants expressed concerns about unintentionally offending, making cultural missteps, or lacking the knowledge needed to navigate beliefs and practices different from their own:
  With no malice or intent, I probably have phrased things wrong or made some cultural gaffes. I think that can sometimes be the issue, is that people would be ignorant to the fact of things that they've done that may not be seen as culturally appropriate.
  – Participant 5, Healthcare professional, Hospital
  I probably could improve on navigating those cultural things and understanding them better myself so then I can navigate them, because unfortunately I'm not always aware of certain cultural kind of beliefs. I'd like to be, but it's sometimes a lot of things to remember.
  – Participant 7, Healthcare professional, Hospital
  I think I consider myself pretty open and approachable and, I want to learn, and I want to do it the right [way]‐ but I don't come from those backgrounds [non‐English speaking background] so how can I possibly know or understand. Absolutely, my white bias is big; I'm always learning, and I think that's where we struggle.
  – Participant 1, Community engagement coordinator, NGO
ii. **Systemic barriers to cultural competence**
  While they are expected to be culturally and linguistically aware, one participant highlighted that the training provided is often minimal. Without regular reinforcement, knowledge can fade over time, making it difficult for staff to consistently apply it in their practice:
  I don't think it is as informative as it could be. I think yes, we are supposed to be all culturally and linguistically aware and we do this kind of online module that's maybe 20 minutes We do that once off. So, we start at the hospital, and we do that one time, it's not an every‐year thing, so it does probably get forgotten a bit. I really try my hardest to respect and be aware of other people's cultures, but there is a lot to probably consider.
  – Participant 7, Healthcare professional, Hospital
iii. **Limited availability and concerns about interpreter quality**
  Healthcare interpreter services helped and hindered the delivery of CVD or stroke prevention education. In the hospital setting, healthcare professionals reported that there is often long wait times and limited availability of interpreter services, especially for high‐demand languages or rare dialects:
  We always try to get an interpreter but that can be extremely difficult. The Healthcare Interpreter Service has very long wait times, and limited number of interpreters. Especially if it's like a rarer dialect, there's a smaller number of interpreters and if it's a high frequency language, especially something like Mandarin or Cantonese in our area because it's such a high frequency language, there's a long, long wait to get interpreters.
  – Participant 4, Healthcare professional, Hospital
  We are very lucky to have really great access to interpreters, but at the same time, we do also get cultures that have languages that aren't very common, like I guess different dialects from Africa which are hard to find interpreters for.
  – Participant 7, Healthcare professional, Hospital
  Participants also noted significant challenges when language barriers intersect with clinical complexity. Although interpreters can assist with communication, their lack of specialised knowledge in managing conditions such as aphasia often limits the effectiveness of support provided:
  I guess with patients who have aphasia and also speak a different language, that's probably the hardest part, I think and that's probably something that we do struggle with a lot. For example, this patient obviously had a low mood, but we also couldn't assess that due to the aphasia plus language barrier as well and trying to be able to use simple words from their language was really difficult, like with an interpreter, for example, because the interpreter doesn't also have the knowledge that we do to be able to navigate aphasia.
  – Participant 7, Healthcare professional, Hospital
  Another participant raised concerns about the accuracy and reliability of interpreter services in clinical settings, noting that some interpreters lacked fluency or native‐level proficiency in the patient's language, which created uncertainty about the quality of communication:
  When I worked in ICU, some translators would come to translate, but I don't quite understand what they're talking about, ‘cause some translator, they're not even a native speaker for that language…
  – Participant 8, Healthcare professional, Hospital
  In situations where professional interpreters are unavailable or wait times are too long, healthcare professionals may turn to digital tools as quick, alternative communication methods:
  …sometimes there's the Google Translate app, which can also assist in the times when, interpreters are not available, and sometimes they feel comfortable to be able to communicate in that sense as well. It's not used very often but it's also used as like a quick option for the patients.
  – Participant 6, Healthcare professional, Hospital
  As this participant describes, they may also rely on ad hoc interpreters, such as family members, bilingual staff, or students, despite knowing these alternatives do not comply with policy standards:
  We couldn't get an interpreter for a full week to even do an assessment. That's best practice and that is the hospital policy but sometimes we will also use family members to do a bit of informal interpretation or even staff or students that speak that language as well. Like I said, it's not best practice and it's not policy but like when you're faced with waiting a week to see where someone's up to or give that information to a family or a patient, you do what you can.
  – Participant 4, Healthcare professional, Hospital
iv. **Lack of culturally relevant and translated resources**
  Another barrier was the limited culturally relevant and translated resources, especially in less common languages, to support healthcare professionals in delivering CVD or stroke prevention education:
  It's just literally having information in their language, there's just not enough or none. Like I can find pretty much everything, I can find it written in Chinese and maybe Turkish and Arabic, but there's lots of other languages that are just not covered. I don't think they cover it. I mean like I said there's supposed to be over 200 different languages spoken in Australia,
  – Participant 2, Healthcare professional, Hospital
  As a result, one participant reported that they have used resources from another country to give to their patients from non‐English speaking backgrounds:
  What I tend to use is a non‐Australian site, it's the stroke.org.uk and they have a much wider variety of languages available in printed form, in much more detailed version. So, I tend to use those.
  – Participant 2, Healthcare professional, Hospital
  Participants felt that having culturally and linguistically diverse staff ensures that CVD and stroke prevention information is not only translated but also framed in a way that respects the beliefs, traditions, and communication styles of different communities:
  Behind the thoughts, behind the behaviour, behind the traditional way, so that's something that I feel really helpful is to have culturally linguistically understanding staff working in the field, which is very important.
  – Participant 13, Health promotion manager, Multicultural Health network
v. **Limited capacity and funding**
  Participants highlighted the challenges related to the limited capacity and funding to meet the educational needs of CALD communities:
  …but we're just so limited with funding and time. There's only me and it's a little bit beyond our capability at the moment… to make those connections and it obviously takes time as well.
  – Participant 1, Community engagement coordinator, NGO
  CALD communities are hungry for information. You know, wherever you go, they say that, when are you coming back? You know, you're working in health, and we are very short staffed everywhere and now covering the whole area. So, we cannot be available every day. But we will say look we will see what we can do, and, in some sessions, you need more experts.
  – Participant 11, Health promotion officer, Multicultural Health network
  The cost of translation is another major limitation. One participant explains that while some resources have been translated, many crucial materials remain unavailable due to the high cost of professional translation services:
  We also have some of our education handouts translated into different languages but that's another barrier as well because it costs money. We have to pay for Healthcare Interpreter Services to translate those resources and often there's not money.
  – Participant 4, Healthcare professional, Hospital

### 3.4 Components of an Ideal Educational Programme

i. **Culturally responsive education**
  There was a strong emphasis on providing education that goes beyond direct translation of content. One participant pointed out that many CVD and stroke prevention resources for CALD communities were translated word‐for‐word but failed to capture underlying sentiments, beliefs and lived experience that shaped how people understand and respond to health information:
  It's not only language to language. It's also around sentiment to sentiment on how we understand their culture and their belief around any of their chronic conditions or health conditions. Let's say when we create any resources for CALD communities most of the time, they are actually translated word to word and does not address the sentiment of a person.
  – Participant 14, Health promotion manager, Multicultural Health network
  Another participant also explained that even when CVD and stroke prevention messages are well‐intentioned, they can fail to resonate with CALD communities if the examples, metaphors, or storytelling techniques do not align with their lived experiences and cultural contexts:
  …if you're someone who isn't part of their culture, and so you can be telling this great story to help them understand something, but you've completely lost them, because the references are not relevant to them at all.
  – Participant 9, Community engagement manager, NGO
  An example of this was provided in relation to healthy eating advice. Participants explained that mainstream dietary advice often reflects an “Anglo approach” to healthy eating, which may not be relevant for all cultural groups:
  Maybe for the eating well component, we need to consider different diets. So obviously people from different backgrounds eat different foods and at the moment it's very much an Anglo approach to eating well. I think that could be an improvement, talking about [South Asian] food and how can you eat better in their context.
  – Participant 1, Community engagement coordinator, NGO
  This was a concern as many individuals seek specific and practical advice on what foods they should eat within their cultural context:
  They often ask, what shall I eat? You cannot just say you need to have low‐cholesterol diet. They will ask, shall I eat fish? Shall I eat meat? I guess different culture[s] have different type of cooking techniques, how they should eat as well. They want something more suitable to their cultural needs… They want more specific information. Like if you say have a healthy diet, what is a healthy diet?
  – Participant 8, Healthcare professional, Hospital
  There was also an emphasis placed on translated resources being reviewed by people with lived experience to ensure they are meaningful, respectful and accessible to those who need them most:
  The first thing I would say is having people from the same language speaking backgrounds to actually go through the document properly. And I would suggest not having just one person go through the document but having at least a number of people – 10 to 15 and not from one sector. So, people who have that condition and who have faced that issue. If they can be a part of proofreading the translated version, then it would give us more idea on their cultural belief because of course, I might learn a new language and if you give me a document. I can read the document, I can say OK, this is not right. This is wrong, but I don't understand the cultural values of that CALD community. I have to be from there to understand their culture and my perspective might differ from another person from the same CALD background.
  – Participant 14, Health promotion manager, Multicultural Health network
ii. **Accessible formats and preferred method of delivery**
  Participants recommended a blended approach including personal narratives, informal discussions, and written materials in accessible formats. This can create a more meaningful and lasting impact on CALD communities' understanding of CVD or stroke prevention and management:
  We also had a video that we showed, and we had that dubbed in other languages. I wish that we had the opportunity to ‐ we had some community stories as well. We didn't get them translated, and if we had the money, I 100 percent would have worked on having some videos, like human stories of real people speaking in their language.
  – Participant 9, Community engagement manager, NGO
  Having stories from their communities within the presentation, more local statistics rather than Australian‐wide perhaps, just making it a bit more customised…
  – Participant 1, Community engagement coordinator, NGO
  It is again probably individual, but I think some form of written education is always good. Whether that is a pamphlet, something emailed, a website someone can go to. Just something written because ‐ like I said, it's hard to remember everything but absolutely always simple language. This is for anyone. CALD or otherwise. Simple language. Diagrams. Pictures.
  – Participant 4, Healthcare professional, Hospital
  They emphasised that traditional lecture‐style presentations may not be the most effective approach. Instead, more interactive and culturally appropriate settings, such as informal group discussions during shared meals, could foster deeper engagement and improve information retention and participation:
  It's really great but the way the presentation's set up, as I said before, it's very much like somebody standing up giving a ‐ there's a PowerPoint presentation. Is that the most appropriate way? I mean, we would love to find out‐ as I said, I think food is important in a lot of these communities. We've heard that perhaps sitting around in a circle and discussing the main points but not doing a formal presentation might be more appropriate and get the message through better, and I think using more stories from their community might be more effective.
  – Participant 1, Community engagement coordinator, NGO
  Some participants recommended using digital technology to deliver CVD and stroke prevention education. However, others felt that while some individuals, particularly those who are familiar with smartphones and the internet, could benefit from online resources, some within CALD communities face significant barriers to digital access and literacy:
  For people that do have access to smart phones, an internet resource would be amazing.
  – Participant 3, Healthcare professional, Hospital
  I don't believe in electronic communication unless they are at a certain level of English and able to use the technology as well.
  – Participant 12, Health promotion officer, Multicultural Health network
  There were concerns older adults, in particular, may struggle with technology, finding it difficult to navigate online platforms, or to distinguish between different messaging apps, or even read notifications as highlighted by these participants:
  Translated information will be a great opportunity for them. But again, technology use is a barrier. If we're talking about online platforms. I know a lot of people that have been in the country for a long time, and they still cannot read WhatsApp notifications. They all get confused between what is WhatsApp, what is Facebook Messenger or the message, they have all of these notifications and emails that are unread because they can't find it.
  – Participant 12, Health promotion officer, Multicultural Health network
  Most are above the age of 65 and they don't have that much skill in technology, and it would be very hard for them to understand what we are saying if it is digital.
  – Participant 14, Health promotion manager, Multicultural Health network
  As such, there was an emphasis on the importance of user‐friendly designs, noting that even native English speakers can find it challenging to navigate online health resources:
  It'd be nice if there was like some sort of centralised resource. I mean I know there is like the Stroke Foundation, obviously they do have the online stuff, but even for me as an English as a first language, I go on their site and sometimes I find it a bit hard to navigate that.
  – Participant 2, Healthcare professional, Hospital
  Participants also stressed the need for trusted, accessible and standardised health resources, ideally developed by reputable organisations to reduce the likelihood of misinformation:
  But I think it would be helpful to have something that is standardised across the state or Australia that you could rely on as a resource, like if, for example, the Stroke Foundation, they created something like that, I think that would be really useful, because you know what it's saying, but it's just in a different language. But if it was off YouTube, you can't rely on it.
  – Participant 7, Healthcare professional, Hospital
iii. **Simple and evidence‐based**
  Participants explained that individuals from CALD backgrounds have varying levels of health literacy shaped by their culture, migration experiences and exposure to different health systems, that could affect their understanding of CVD and stroke prevention information:
  Health literacy can be different as well especially if people have migrated from a different country or are refugees.
  – Participant 4, Healthcare professional, Hospital
  I think it's the level of understanding and also the language used, and the terminology used and their general health literacy in that area. I guess when I did come across them, a lot of them were not familiar with the actual terminology and the language.
  – Participant 6, Healthcare professional, Hospital
  One participant highlighted that literacy within a person's native language should also be considered when developing educational resources. Older generations in some communities may not have had access to formal education and may be unable to read materials even in their native language:
  Even some small proportion of patients in [East Asia], the older generation, they cannot read in the [language], like my great‐grandmother. But she's already 80 or 90 years old because in the old days, they are not well educated. So, if you're thinking of that factors, if you give patients a version in [language] to read, maybe they cannot read as well.
  – Participant 8, Healthcare professional, Hospital
  As such, having concise, simple and easy to understand CVD and stroke prevention information would also accommodate differences in health literacy:
  We don't use a lot of medical terminology or medical jargons [sic]. Our staff are well trained in that area. We need to always keep it simple and not to bombard them with a lot of information. Because these people, sometimes they don't understand what you're talking about. So sometimes you have to be very, very down to earth.
  – Participant 11, Health promotion officer, Multicultural Health network
  One participant also explained that basic, easy to understand information especially when delivered in community languages is often more effective and sustainable than lengthy, complex resources that may quickly become outdated:
  I would just love seeing really simple, easy to understand. [sic] Simple can sometimes be misconstrued. But really easy to understand information in language, rather than spending a lot of money on, trying to translate big pieces of resources. Because they go out of date very, very quickly.
  – Participant 9, Community engagement manager, NGO
iv. **Ongoing reinforcement of educational content**
  Health education and support should not be a one‐time event. A recurring theme was the need for regular reminders, follow‐ups, and reinforcement to ensure lasting impact:
  A regular reminder is also important, a [sic] bimonthly simple information. I think a lot of people already know that junk food is junk food, and if you've got high blood pressure, this is what you're going to do. Diabetes is not good for you. I think a lot of people already know that. But it's just that support that they need, you know, amongst all the other life events that's happening. So, like monthly reminder or bimonthly reminder saying, have you done this…
  – Participant 10, Health promotion officer, Multicultural Health network
v. **Better engagement of family, caregivers and community**
  The role families play in aiding recovery is important but complex. They need be supportive and while empowering independence. This balancing act can be easily upset by family members' personal beliefs:
  That's the thing you see a lot, is that family members being present is a wonderful thing but really ‐ I think the message that people need [to understand]is stroke patients need to be doing things ‐ practising ‐ to enable their recovery, and family members not necessarily knowing what is the best way to help and often the thought around helping is by doing everything when that's not necessarily what we want.
  – Participant 5, Healthcare professional, Hospital
  I've seen quite a few patients that have had really awful outcomes with stroke because their families have said no, you shouldn't be taking that medication which is mind‐blowingly awful when they come in and they've had a stroke and it's because of medication non‐compliance.
  – Participant 3, Healthcare professional, Hospital
  Participants felt that there is a need to better engage family in the education process whether through in‐person discussions, multilingual resources, or culturally tailored support networks to foster a stronger, more informed support system:
  Ideally my role is to try and get their family members there for that conversation and it's about building that conversation with those support people for that person and about gathering their needs. It would be engaging family members as well I think could be really impactful. Yeah, which you would do by giving them something to take home or something they can reference when you're not with them.
  – Participant 3, Healthcare professional, Hospital
  They further explained that when families are informed and equipped with the right knowledge, they can provide better support and reinforce key health messages at home:
  Perhaps providing education to the family, the carers, for them to provide assistance for the patient and setting them up with services or culturally appropriate services for them to be supported in their community and within their environment, so that they can be more confident. And further develop and improve physically after discharge from hospital.
  – Participant 6, Healthcare professional, Hospital
  Participants also emphasised the value of informing people about services they can access to support communication with healthcare professionals and self‐management:
  And I think it's also important to empower them. Right at the admission say, you can access interpreter service, if at any time you don't understand. Once you receive CALD patients, empower them, ‘You can access this support. Even if you have language barriers, it's okay to say, I need help to access the support I need.’
  – Participant 8, Healthcare professional, Hospital
  The value of the broader community in encouraging health‐seeking behaviours was also highlighted. Participants explained that when discussion about prevention such as heart health checks become normalised within the community, individuals are more likely to seek care and establish ongoing relationships with healthcare professionals:
  We would encourage them to speak to their friends and their community, which then built up another sort of confidence around the fact that this is now being talked about within their community, is going and getting a heart health check, and having a GP that you have a relationship with…
  – Participant 9, Community engagement manager, NGO
  Building trust and fostering relationships was considered key to effective health education within CALD communities. This participant emphasised that engagement goes beyond simply delivering information. When individuals feel connected to a trusted person or space, they are more likely to return, engage and absorb critical health information:
  But as you work with the community, it's just like building this relationship. Again, relationship with the community is very important. This is coming from [Health Promotion officer] working with refugee communities. OK, so having been there speaking to them, give them a chance to meet them [health workers] as a person developing this network because they need these social events. Kind of developed sense of belonging, and that feels safe to that place or to that person who deliver the information. Then they feel they want to listen more. I want to attend more, more of those sessions. I think that's what helped me to deliver.
  – Participant 12, Health promotion officer, Multicultural Health network
vi. **Codesign is key to effective education**
  Genuine codesign and partnership are critical for an effective approach to health education. A common pitfall in Australia's approach is assuming what CALD communities need rather than actively engaging them to shape solutions:
  So, I think that there needs to be a lot of feedback from people who are culturally and linguistically diverse. Australia has a habit of saying well this is what they need so let's do that, instead of what do you need and how can we help? You [community] help us structure it. I do feel like there[‘s] definitely lots that could be done to educate people before it's a problem.
  – Participant 3, Healthcare professional, Hospital
  Another participant emphasised the importance of localised and community‐specific strategies taking into consideration the unique preferences of different cultural groups for health information delivery:
  I think bottom up ‐ definitely more consultation, codesign more localised strategies; so how do the [South Asian] community want to be communicated to versus the Arabic community, or the African community. If there was endless money and time and we could have heaps of staff and employ one person from each, CALD group we're trying to focus on, that would be amazing.
  – Participant 1, Community engagement coordinator, NGO

## 4 Discussion

This study highlights the importance of culturally tailored health education that is co‐developed with communities, delivered in accessible languages and formats, and built on trust. Although challenges like interpreter shortages, limited funding and inadequate access to cultural training remain, involving families, empowering individuals, providing regular reinforcement of educational content and adapting approaches to community needs have the potential to significantly improve the reach and effectiveness of CVD and stroke prevention efforts.

The complexity of delivering health education to CALD communities due to cultural and language barriers has been widely documented [19, 20]. Similar to previous studies [21, 22, 23], our findings demonstrate the difficulties experienced by healthcare professionals when navigating cultural health beliefs and differences in worldviews that could be partly reduced if more healthcare professionals had cultural humility. Cultural humility goes beyond simply understanding other cultures (as in cultural competence) [24]; it involves recognising one's inherent biases and embracing a lifelong commitment to learning and working respectfully with diverse communities [25, 26]. Working with humility reduces the risk of stereotyping, stigmatising and ‘othering’ people from CALD communities which fosters implicit racist attitudes and behaviours [24, 27]. In line with previous studies [22, 28, 29], participants emphasised the importance of increasing CALD representation in the health workforce to ensure cultural safety.

Access to interpreter services and broader resource constraints, including limited funding, staff shortages, and the high cost of translating materials remain a challenge. Although digital tools such as Google Translate and artificial intelligence technologies present a promising, low‐cost solution for facilitating communication and rapid translation, their usefulness is largely confined to basic interactions [30, 31]. Significant concerns persist regarding accuracy, and issues related to privacy and confidentiality [32, 33].

There were numerous suggestions for developing a culturally appropriate education programme. A key theme, consistent with other studies [34, 35, 36] was providing translated content that is simple and culturally relevant. Poor health literacy directly affects the comprehension of health information [37, 38], which highlights the value of plain language and audio‐visual resources [29], and recognising that Western medical concepts may not readily translate across cultures [39]. Cultural beliefs influence how people perceive health and illness and interpret health information [40], reinforcing the need for culturally tailored information that enable informed decisions about cardiovascular health [41]. Culturally tailored programmes can enhance the understanding of disease among CALD communities and support sustained healthy lifestyle changes and treatment adherence [22, 42]. However, the evidence on the effectiveness of these programmes for CVD and stroke prevention remain inconclusive [43].

Tailoring delivery methods and engaging family and community were also considered important. Similar to other studies [28, 29, 44], participants called for simple written resources, story‐telling and informal educational formats, like small group discussions over food. These were viewed as more impactful and accessible than traditional lecture‐style presentations. Sharing food is key in many CALD communities to foster connection and inclusion [45]. While online resources were seen as promising for those comfortable with using technology, the concern that older adults in CALD communities face significant challenges in navigating digital tools has been identified before as problematic [46]. The core digital skills needed for technology use were often limited and were observed across all cultural groups and ages and in a significant minority (13/34) of included studies [46].

Current findings also highlight the importance of involving families and the wider community in education efforts to ensure they understand how to provide appropriate support. Some CALD communities' have a collective identity, where families have different expectations around individual autonomy and independence compared to mainstream families [47]. As family plays a central role in brokering health information and providing support among CALD communities [48], facilitating conversations that include the individual and their support networks were highlighted as key in promoting shared understanding and encouraging families to reinforce key health messages at home. Further, fostering peer‐led education and encouraging discussions within communities around preventative health checks were also seen as effective ways to increase engagement and shift health norms. Grassroots, community‐led, bottom‐up approaches to education that involve CALD communities are key to shaping the content and delivery of CVD and stroke prevention educational programmes. This is consistent with the literature [49, 50], in which codesign improved cultural relevance and fostered trust and engagement.

Research to evaluate the effectiveness of culturally tailored CVD and stroke prevention programmes is needed. While proposed as ideal, we do not know whether educational prevention programmes co‐created with community will ensure cultural safety, relevance, and accessibility, nor whether adaptable, scalable models can be implemented across different cultural groups and settings, or if they are cost effective. The next phase of the study will focus on co‐designing an educational behaviour change programme in collaboration with people with lived experience of CVD and stroke, healthcare professionals, and partners from nongovernment and community organisations. This phase will also include evaluation of the feasibility and acceptability of the programme. Fostering cultural humility rather than simply cultural competence in healthcare education and service delivery is ideal, but ways to do this effectively are untested [51, 52]. There is also a need to include interpreters in the development of programmes by working in partnership with CALD communities to ensure accessibility and cultural relevance.

### 4.1 Strengths and Limitations

One of the main limitations of this study was the use of convenience sampling. Our sample was limited to healthcare professionals and representatives of key cardiovascular and stroke NGOs and multicultural health networks who agreed to participate, which raises the possibility that their accounts may be biased and not all concerns and opinions about CVD and stroke prevention education were mentioned. However, the broad range of views and perceived issues that were raised are consistent with other findings reported in the literature. The other limitation was the lack of representation from primary health care. They play a crucial role in CVD and stroke prevention by providing essential services such as heart health checks, identifying and managing CVD risk factors and promoting healthy lifestyles [53]. The strength of this study is the inclusion of participants from multiple States, representatives from key CVD and stroke organisations and healthcare professionals and health promotion officers from CALD backgrounds. The sample facilitated broad and diverse perspectives, which potentially increases the generalisability of the findings.

## 5 Conclusion

The findings highlight the importance of culturally responsive health education that is co‐designed with communities, delivered in preferred languages and formats, and supported by trust‐building relationships. While system barriers such as interpreter shortages and limited funding persist, engaging families, empowering individuals, tailoring delivery methods, and embracing community‐specific strategies offer clear pathways for improving engagement and reach of CVD and stroke prevention education. Incorporating these components, particularly through codesign and sustainable resource development, will be essential in ensuring equitable cardiovascular outcomes for CALD communities.

## Author Contributions

**Sabine M. Allida:** conceptualisation, funding acquisition and supervision (lead), investigation (equal), formal analysis (equal), writing – original draft (lead), writing – review and editing (equal). **Della Maneze:** investigation, formal analysis and writing – review and editing (equal). **Scott William:** formal analysis and writing – review and editing (equal). **Maree Hackett:** funding acquisition and writing – review and editing (equal). **Caleb Ferguson:** funding acquisition and writing – review and editing (equal).

## Ethics Statement

The study was approved by the Western Sydney Local Health District (WSLHD) Human Research Ethics Committee, New South Wales, Australia (ETH01328; 31 July 2023).

## Consent

All participants provided electronic informed consent to participate.

## Conflicts of Interest

The authors declare no conflicts of interest.

## Declaration Statement on the Use of Artificial Intelligence

During the preparation of this work, the authors used OpenAI ChatGPT to edit and improve the readability of the manuscript. The authors reviewed and edited the content and take full responsibility for the content of the publication.

## Supporting information

Supplementary_COREQ_Checklist.

## Data Availability

The data that support the findings of this study are available on request from the corresponding author. The data are not publicly available due to privacy or ethical restrictions.
